# Cancer Screening in Afghanistan: A Narrative Review of Feasibility, Barriers, and Evidence-Based Strategies for Low-Resource Settings

**DOI:** 10.7759/cureus.101346

**Published:** 2026-01-12

**Authors:** Mahjabin Shahid, Mohammad Zahid Omerzad, Sayed Sahil Hashemi, Nafisa Hamidi, Humaira Sadat Sultany, Mohammad Yousuf Sultani, Hashmat Ullah Faizi, Mohammad Ibrahim Sultani, Samira Ali, Nilab Afzalzada, Aseruddin Wafi, Farid Farhikhta

**Affiliations:** 1 Faculty of Medicine, Ariana University, Kabul, AFG; 2 Faculty of Medicine, Kabul University of Medical Sciences, Kabul, AFG; 3 Department of Medicine, Northern Hospital, Melbourne, AUS; 4 Department of Internal Medicine, Bamyan Provincial Hospital, Bamyan, AFG; 5 Department of General Medicine, Kabul University of Medical Sciences "Abu Ali Ibn Sina", Kabul, AFG; 6 Department of Dentistry, Stomatology National and Teaching Hospital, Kabul, AFG; 7 Department of General Practice, Nangarhar Medical Faculty, Nangarhar University, Jalalabad, AFG; 8 Faculty of Medicine, Ankara Yıldırım Beyazıt University, Ankara, TUR; 9 Department of Obstetrics and Gynecology, Isteqlal Hospital, Kabul, AFG; 10 Faculty of Medicine, Khatam Al-Nabieen University, Kabul, AFG; 11 Department of Internal Medicine, Balkh Medical University, Balkh, AFG

**Keywords:** afghanistan, breast cancer, cancer screening, cervical cancer, healthcare infrastructure, low-resource settings, narrative review, population-based cancer registry, visual inspection with acetic acid

## Abstract

Afghanistan experiences a substantial cancer burden within the South Asian Association for Regional Cooperation region, with poor outcomes largely linked to delayed diagnosis and limited access to diagnostic and treatment services. The national cancer profile is dominated by malignancies commonly observed in low-resource settings, including cancers of the breast, gastrointestinal tract, and cervix. This narrative review examines the limitations of commonly used cancer screening modalities, the systemic and sociocultural barriers to their implementation in Afghanistan, and feasible strategies for early cancer detection in resource-constrained environments. Conventional screening approaches, such as endoscopic procedures, imaging-based screening, and cytology-based tests, face significant challenges related to infrastructure, workforce capacity, affordability, and accessibility, limiting their widespread use. Screening for less prevalent cancers is discussed in the context of uncertain feasibility and limited applicability in low-resource health systems. Afghanistan’s healthcare system continues to face structural constraints, including fragmented oncology services, limited diagnostic capacity, and the absence of coordinated screening frameworks. These challenges are further compounded by sociocultural factors such as low health awareness, stigma, gender-related barriers, and financial hardship, all of which reduce screening uptake and delay care-seeking. In addition, weaknesses in health information systems hinder effective cancer surveillance and planning. This narrative (non-systematic) review emphasizes pragmatic, low-cost approaches to cancer screening and early diagnosis that can be integrated into existing primary healthcare services, highlighting frontline healthcare worker training and community engagement as realistic pathways to improving early detection under severe resource limitations.

## Introduction and background

Afghanistan faces a substantial and growing cancer burden, with some of the highest age-standardized incidence and mortality rates in the South Asian Association for Regional Cooperation (SAARC) region. According to the Global Cancer Observatory and the 2024 SAARC series, the age-standardized incidence rate (ASIR) in Afghanistan is 103.6 per 100,000 for males and 110.7 per 100,000 for females, while the age-standardized mortality rate (ASMR) is 81.0 per 100,000 for males and 77.5 per 100,000 for females. These rates are among the highest in the region, exceeded only by Sri Lanka for incidence, and are notably higher than the global average for low- and middle-income countries (LMICs) [1-3]. The mortality-to-incidence ratio (MIR) in Afghanistan is also elevated, reflecting late-stage presentation and limited access to effective treatment, with an MIR of approximately 0.75 for some cancer types, compared to a global MIR of 0.49 [1,4].

The most common cancers in Afghanistan, as identified by hospital-based studies and regional modeling, are breast, esophageal, stomach, colorectal, and lymphoid malignancies in both sexes, with cervical cancer also prominent among women [1,5]. Breast cancer accounts for approximately 45.8% of female cancer cases, making it the leading cancer among Afghan women, while esophageal cancer is the most common in men (21.8% of male cases) and the second most common in women (12.5%) [5]. Stomach cancer is the second most common in men (12.2%) and also present in women (2.6%). Colorectal cancer represents 8.6% of male and 4.8% of female cases, and non-Hodgkin lymphoma accounts for 9.4% of male and 4.7% of female cases [5]. Although informative, this evidence is derived from a single tertiary hospital in Kabul and may not fully represent national cancer prevalence patterns. Cervical cancer, though underreported due to limited screening, is among the top five incident and fatal cancers in Afghan women [1,3].

Therefore, this narrative review aims to identify context-appropriate, evidence-based cancer screening strategies that are feasible within Afghanistan’s current health system and resource constraints.

Table 1 provides a detailed breakdown of the incidence, prevalence, and mortality of major cancer types in Afghanistan, highlighting the high burden and poor outcomes across the spectrum of malignancies. This table is essential for understanding the quantitative landscape of cancer in Afghanistan and for prioritizing screening and control efforts.

**Table 1:** Incidence, prevalence, and mortality of major cancer types in Afghanistan ASIR, age-standardized incidence rate; ASMR, age-standardized mortality rate; MIR, mortality-to-incidence ratio

Cancer type	Estimated incidence (ASIR per 100,000)	Prevalence (hospital-based %)	Estimated mortality (ASMR per 100,000)	MIR	References
Breast (female)	110.7 (female, all cancers)	45.8% of female cases	High; leading cause of death in women	High	[1-3]
Esophagus	21.8% (male), 12.5% (female)	21.8% male, 12.5% female	High; leading cause of death in men	High	[1-3]
Stomach	12.2% (male), 2.6% (female)	12.2% male, 2.6% female	High; top cause of death in men	High	[1,3,4]
Colorectal	8.6% (male), 4.8% (female)	8.6% male, 4.8% female	Moderate to high	Moderate to high	[1,3]
Non-Hodgkin lymphoma	9.4% (male), 4.7% (female)	9.4% male, 4.7% female	Moderate	Moderate	[3,5]
Cervical (female)	Not specified; among the top 5 in the region	1.9% of female cases	High; major cause of death in women	High	[1-3]
Lung	2.7% (male)	2.7% male	Moderate to high	High	[1,3]
Liver	3.2% (male), 2.6% (female)	3.2% male, 2.6% female	Moderate	High	[1,3,4]
Ovary (female)	Not specified	3.8% of female cases	Moderate	Moderate	[3]

Comparing Afghanistan to other SAARC countries, Afghanistan’s ASIR and ASMR are among the highest, and its MIR is elevated, indicating poorer outcomes and higher case fatality. For example, Bangladesh has an ASIR of 120.8 for men and 89.5 for women, while Nepal has the lowest ASMR at 55.3 per 100,000 [1,4]. The high burden and mortality in Afghanistan are attributed to decades of political instability, underdeveloped healthcare infrastructure, and lack of organized screening and early detection programs [1,6].

## Review

Methodology

This is a narrative review that synthesizes existing literature on cancer screening modalities, implementation barriers, and feasible strategies for low-resource settings, with specific application to Afghanistan. Unlike systematic reviews that follow structured protocols such as Preferred Reporting Items for Systematic reviews and Meta-Analyses (PRISMA), narrative reviews provide a comprehensive, expert-driven analysis of a broad topic, allowing for critical interpretation and integration of diverse evidence sources, including epidemiological data, clinical trials, implementation science studies, and policy documents.

Literature Search Strategy

The literature search was conducted using PubMed, Google Scholar, and specialized databases, including the Global Cancer Observatory (GLOBOCAN), with a focus on publications from 2015-2025 to capture recent evidence. Search terms included combinations of “cancer screening”, “Afghanistan”, “low-resource settings”, “SAARC”, “visual inspection with acetic acid”, “cervical cancer screening”, “breast cancer screening”, “population-based cancer registry”, and “cancer control in low-income countries”. Additional sources were identified through reference lists of key articles and regional cancer control reports from authoritative organizations (e.g., WHO technical reports, GLOBOCAN fact sheets, and SAARC policy documents).

Inclusion criteria encompassed studies and reports addressing (1) cancer epidemiology and burden in Afghanistan and comparable SAARC countries; (2) performance characteristics and limitations of cancer screening modalities; (3) barriers to screening implementation in low-resource settings; (4) evidence-based screening strategies appropriate for resource-constrained environments; and (5) cancer registry and surveillance systems in conflict-affected regions. Both peer-reviewed research and gray literature from authoritative organizations such as WHO and Disease Control Priorities project were included.

The narrative review approach was selected as most appropriate for this topic because (1) the research question requires synthesis of heterogeneous evidence types, including epidemiological data, screening test performance, implementation barriers, and policy recommendations; (2) the Afghanistan context necessitates expert interpretation of how global evidence applies to a unique setting with severe resource constraints and ongoing conflict; and (3) the goal is to provide actionable, context-specific recommendations rather than a quantitative meta-analysis of screening effectiveness. This methodology allows for critical analysis of the complex interplay between healthcare infrastructure, sociocultural factors, and screening feasibility in Afghanistan.

Limitations of cancer screening modalities

Major Cancer Types

Screening for major cancers in Afghanistan is constrained by the intrinsic limitations of available modalities and the severe deficits in health system capacity. For colorectal cancer, colonoscopy is considered the gold standard, with a sensitivity of 0.89 for detecting adenomas ≥6 mm and a specificity of 0.89, but it is invasive, costly, and associated with risks such as perforation (3.1 per 10,000 procedures) and major bleeding (14.6 per 10,000 procedures) [7]. Patient compliance is limited by the need for bowel preparation, sedation, and time off work, and accessibility is restricted by the lack of specialized equipment and trained personnel [8-10]. Noninvasive tests such as the fecal immunochemical test (FIT) have a pooled sensitivity of 0.74 and specificity of 0.94 for cancer detection, while the guaiac-based fecal occult blood test has lower sensitivity (0.50-0.75) but high specificity (0.96-0.98) [7]. Multi-target stool DNA testing offers higher sensitivity (0.93) but lower specificity (0.85) compared to FIT [7]. Computed tomography colonography provides similar sensitivity to colonoscopy for lesions ≥6 mm but exposes patients to ionizing radiation and may lead to incidental findings that require further workup, increasing cost and risk [7,9]. Adherence to repeated screening and follow-up after positive tests is a major challenge, and effectiveness is highly dependent on patient compliance [10,11].

For breast cancer, mammography is the primary screening modality, with sensitivity ranging from 75% to 90% depending on age and breast density, and high specificity [12,13]. However, its performance is reduced in women with dense breast tissue, leading to false negatives. Overdiagnosis and overtreatment are significant concerns, as mammography can detect indolent lesions that may never become clinically significant, exposing patients to unnecessary biopsies and treatments [13]. Accessibility and cost vary widely, with lower availability in low-resource settings. Patient compliance is generally high, but discomfort during the procedure and anxiety related to false positives can reduce adherence [12,14]. The risk of radiation exposure is low but not negligible, especially with repeated screening over many years [13].

Cervical cancer screening relies on the Papanicolaou (Pap) test and human papillomavirus (HPV) testing. The Pap test has moderate sensitivity (about 50-75%) but high specificity, while HPV testing offers higher sensitivity (over 90%) but lower specificity, leading to more false positives and potential overtreatment [12,14]. Visual inspection with acetic acid (VIA) is a low-cost alternative with acceptable sensitivity and specificity and is recommended for low-resource settings [15,16]. Accessibility is limited in regions without organized screening programs, and cost can be a barrier in low-income settings. Patient compliance is generally good, but cultural factors and lack of awareness can reduce participation, particularly in resource-limited countries [17-19].

Lung cancer screening with low-dose computed tomography (LDCT) is recommended for high-risk individuals (typically aged 55-74 years with a significant smoking history). LDCT has higher sensitivity than chest radiography but lower specificity, resulting in a substantial rate of false positives and unnecessary follow-up procedures [20,21]. The risk of cumulative radiation exposure is a concern, especially with annual screening. Accessibility is limited by the need for advanced imaging equipment and trained radiologists, and cost is high relative to other screening modalities. Patient compliance is low, with less than 4% of eligible high-risk individuals undergoing LDCT in the United States and even lower rates in low-resource settings [12].

Prostate cancer screening with prostate-specific antigen (PSA) testing has limited sensitivity and specificity, leading to high rates of false positives and overdiagnosis of indolent cancers that may never cause harm [13,20,21]. The balance of benefits and harms is uncertain, and major guidelines recommend informed or shared decision-making rather than routine screening for all men [12,21]. Risks associated with transrectal biopsy include infection and, rarely, septicemia [13].

Less Common Cancers

Screening for less common cancers, such as ovarian, liver (hepatocellular carcinoma), sarcoma, and testicular cancers, presents distinct limitations. For ovarian cancer, large randomized controlled trials (PLCO and UKCTOCS) have demonstrated that annual screening with transvaginal ultrasound and/or serum CA-125 testing does not reduce ovarian cancer mortality in average-risk, asymptomatic women [22-24]. The U.S. Preventive Services Task Force, the American College of Obstetricians and Gynecologists, and the American Cancer Society all recommend against routine screening for ovarian cancer in the general population, citing insufficient sensitivity and specificity, low positive predictive value (4.6-10.8%), and a high rate of false positives leading to unnecessary surgical interventions and associated morbidity [22,24]. Even in high-risk populations, screening has not shown a mortality benefit [24].

For liver cancer (hepatocellular carcinoma), screening is recommended only for high-risk populations, such as individuals with cirrhosis or chronic hepatitis B infection. The standard modality is abdominal ultrasound every six months, with or without serum alpha-fetoprotein (AFP) measurement. Ultrasound alone has a sensitivity of approximately 72% and a specificity of 94% for HCC detection, but sensitivity drops to 53% for resectable tumors. AFP at a cutoff of 20 ng/mL yields a sensitivity of 60% and specificity of 84%, but at higher cutoffs (200 ng/mL), sensitivity falls to 36% while specificity rises to 99%. Combining ultrasound and AFP increases sensitivity to 96% but reduces specificity to 85%, resulting in more false positives and unnecessary follow-up [25-27]. However, the diagnostic accuracy is suboptimal, and there is a paucity of data supporting the survival benefit of HCC screening in resource-limited settings [25,26].

Sarcomas are a heterogeneous group of malignancies, and there are no established population-based screening modalities for either soft tissue or bone sarcomas. Diagnosis typically relies on clinical suspicion, physical examination, and imaging (plain radiographs, ultrasound, CT, and MRI), followed by biopsy. In basic resource settings, such as Afghanistan, initial diagnosis and staging usually occur outside formal oncology centers, often through primary care providers or general surgeons. Access to advanced imaging and pathology is extremely limited, and referral pathways are poorly developed [28].

Testicular cancer is most common in young men, and while it is highly curable when detected early, there is no evidence supporting population-based screening. The Cochrane review found no randomized controlled trials evaluating the effectiveness of screening (physician or self-examination) in reducing testicular cancer-specific mortality. Major organizations recommend against routine screening due to the low incidence and favorable outcomes with prompt diagnosis and treatment. Scrotal ultrasound is highly sensitive and specific (>90%) for detecting testicular lesions, but its use as a screening tool is not recommended [29,30].

Across all these less common cancers, the limitations of screening modalities are magnified by Afghanistan’s severe healthcare infrastructure deficits, resource constraints, and sociocultural barriers. There is a lack of organized screening programs, minimal access to diagnostic equipment and trained personnel, and poor referral systems. The consensus in the literature is that population-based screening for ovarian, liver, sarcoma, and testicular cancers is not recommended in low-resource settings, including Afghanistan, due to poor test performance, lack of survival benefit, and the risk of harm from false positives and unnecessary interventions [31-33].

Barriers to effective screening implementation

The implementation and effectiveness of cancer screening programs in Afghanistan are profoundly shaped by the interplay of healthcare infrastructure limitations, resource constraints, and sociocultural factors. Afghanistan’s healthcare infrastructure is severely underdeveloped, particularly for cancer care and screening. The country has only basic oncology services, which are largely restricted to Kabul, with minimal capacity for advanced diagnostics, radiology, pathology, and treatment modalities such as radiotherapy and chemotherapy. There are only 0.5 public cancer centers per 10,000 cancer patients, and no national cancer management guidelines or organized screening programs for breast, cervical, or colorectal cancer exist [1,6,34].

Resource limitations in Afghanistan are multifaceted, encompassing financial, human, and technological domains. Government spending on health care is low, and public health insurance schemes are either insufficient or nonexistent, leading to high out-of-pocket costs for patients. Essential medical equipment, such as mammography units, radiotherapy machines, and advanced laboratory facilities, is in short supply, resulting in long waiting times and delayed or missed diagnoses. The cost of cancer screening modalities is prohibitive for most Afghans, especially in the absence of subsidized programs. The shortage of trained health professionals is exacerbated by limited training opportunities and the migration of skilled workers to higher-income countries [1,6,31].

Sociocultural barriers are pervasive and significantly impede the uptake and effectiveness of cancer screening programs in Afghanistan. Low health literacy and the absence of organized public health education campaigns contribute to poor awareness of cancer risk factors, symptoms, and the benefits of early detection. Studies among Afghan refugee women have demonstrated extremely low awareness of breast cancer warning signs and risk factors, with virtually no utilization of screening services; these findings are likely reflective of the situation within Afghanistan itself [17-19,34]. Cultural stigma surrounding cancer, particularly breast and cervical cancers, leads to embarrassment, fear, and reluctance to seek screening. Gender norms and inequities further restrict access, as many women prefer female healthcare providers for intimate examinations, yet female providers are scarce. Familial and spousal disapproval, especially in conservative and rural areas, can prevent women from accessing screening altogether [19,35,36].

Financial constraints and geographic barriers also play a major role. Out-of-pocket costs for screening, travel expenses, and lost wages deter participation, particularly among low-income and rural populations. The challenging terrain and poor transportation infrastructure make it difficult for women to reach health facilities, and long travel distances are a well-documented impediment to screening uptake [6,36]. The lack of health insurance and public funding for screening programs means that even low-cost interventions like VIA and clinical breast examination (CBE) are not universally accessible.

At the health system level, poor coordination between primary, secondary, and tertiary care leads to inefficiencies in referral and follow-up. Fragmented data systems and underdeveloped cancer registries hinder the ability to track screening coverage, monitor outcomes, and evaluate program effectiveness. The absence of national cancer control policies and guidelines for screening results in inconsistent implementation and poor quality assurance [37-41].

Feasible and evidence-based screening strategies

Given Afghanistan’s resource and infrastructure constraints, the development of context-appropriate, feasible cancer screening strategies is essential. The evidence base, including recent regional analyses and global recommendations, consistently emphasizes that population-wide, high-technology screening programs, such as mammography for breast cancer, colonoscopy for colorectal cancer, or PSA testing for prostate cancer, are not feasible or cost-effective in low-resource settings like Afghanistan [31,42]. Instead, the focus should be on pragmatic, low-cost, and scalable interventions that can be integrated into existing health services, with an emphasis on early diagnosis, community education, and linkage to treatment.

For cervical cancer, opportunistic or targeted screening using VIA is the most feasible and effective approach in low-resource settings. VIA is inexpensive, requires minimal equipment, and can be performed by trained frontline health workers. The Disease Control Priorities, 3rd Edition (DCP-3) essential package specifically recommends opportunistic cervical screening with VIA for LMICs, including Afghanistan, as it can detect precancerous changes that are amenable to immediate, low-cost treatment, often in a single visit (the “screen-and-treat” approach) [16,39]. Randomized trials in India and sub-Saharan Africa have demonstrated that VIA-based screening, especially when combined with immediate cryotherapy for eligible lesions, can reduce cervical cancer incidence and mortality by 25-35% over 7-12 years [43]. While HPV testing is more sensitive, it is currently less feasible due to cost and logistical barriers but may become a future option as affordable rapid tests become available [44-46].

For breast cancer, mammography-based screening is not feasible due to cost, infrastructure, and workforce limitations [15,31,43]. CBE may be considered as a pragmatic alternative, but its effectiveness in reducing mortality remains uncertain, and quality assurance is critical [31,43]. The highest-yield strategy for Afghanistan is to improve early diagnosis through community education, awareness campaigns, and training of frontline health workers to recognize and promptly refer women with breast symptoms [34,43]. Early diagnosis initiatives should be linked to accessible diagnostic and treatment services, with efforts to reduce patient and health system delays [42,43].

For colorectal cancer, population-based screening with colonoscopy or FIT is not currently feasible due to resource constraints [15,31,42]. The literature suggests that only LMICs with a rising trend of colorectal cancer and adequate resources should consider demonstration projects using FIT [31]. For Afghanistan, the priority should be on raising awareness of colorectal cancer symptoms among the public and healthcare providers and strengthening referral pathways for symptomatic individuals [42]. Opportunistic screening of high-risk individuals may be considered in the future as resources allow, but it is not currently recommended as a population-wide strategy [31,42].

For oral cancer, opportunistic screening among habitual tobacco and/or alcohol users using oral visual examination may be considered, particularly in high-risk groups [15,31]. However, the primary focus should remain on primary prevention through tobacco and alcohol cessation programs, as these have the greatest impact on reducing oral cancer incidence [15,31].

There is no evidence to support population-based screening for prostate, lung, stomach, or ovarian cancer in Afghanistan or similar LMICs, given the lack of cost-effective, feasible modalities and the risk of overdiagnosis and overtreatment [15,31-33]. For these cancers, the emphasis should be on early diagnosis of symptomatic cases, risk factor reduction (e.g., tobacco control for lung cancer), and strengthening diagnostic and treatment capacity [42].

Table 2 provides a concise overview of feasible cancer screening strategies for Afghanistan, including recommended modalities, rationale, and references. These feasibility recommendations synthesize the policy-relevant implications of the preceding analysis and are intended to guide clinical and health policy decision-making in resource-constrained settings.

**Table 2:** Feasible cancer screening strategies and recommendations for Afghanistan CBE, clinical breast examination; FIT, fecal immunochemical test; OVE, oral visual examination; VIA, visual inspection with acetic acid

Cancer type	Recommended screening strategy	Rationale/notes	References
Cervical	VIA-based opportunistic screening; screen-and-treat with cryotherapy	Most feasible, low-cost, effective; can be performed by trained health workers; reduces mortality	[1-5]
Breast	Early diagnosis via community education, awareness, and prompt referral; CBE as an adjunct	Mammography not feasible; CBE effectiveness uncertain; focus on awareness and early diagnosis	[1,2,4-6]
Colorectal	Symptom-based early diagnosis; FIT demonstration projects if resources allow	Population screening not feasible; focus on awareness and referral for symptoms	[1,2,4]
Oral	Opportunistic OVE in high-risk (tobacco/alcohol users); primary prevention	Screening in high-risk groups; prioritize tobacco/alcohol cessation	[1,2]
Prostate, lung, stomach, and ovarian	No population-based screening recommended	Not feasible or cost-effective; focus on early diagnosis and risk factor reduction	[1,2,4]

Integration of cancer screening with existing primary care and maternal health services is strongly recommended. Training primary care providers, including midwives and community health workers, in VIA for cervical cancer and CBE for breast cancer, and equipping these centers with the necessary supplies can increase coverage and reduce missed opportunities [39-41]. Maternal health visits, such as antenatal and postnatal care, provide critical opportunities to offer cancer screening and education, as women are more likely to access these services than standalone cancer screening clinics [1,6,15]. Community education and demand generation are essential to increase awareness and reduce stigma and should be tailored to rural and underserved populations using mass media, mobile health applications, and telemedicine [36,39].

Quality assurance mechanisms, including supervision, mentorship, and the use of technology (e.g., smartphone-based VIA and telemedicine support), are critical to ensure the effectiveness and safety of integrated screening programs [39,40]. Digital registries facilitate monitoring and evaluation, enabling health systems to track participation, outcomes, and follow-up and to identify gaps for continuous improvement [38,45].

Cancer registry, surveillance, and data gaps

Accurate estimation of cancer burden and mortality in Afghanistan is critically hampered by substantial gaps and challenges in the country’s cancer registry and reporting systems. Afghanistan lacks a comprehensive, population-based cancer registry (PBCR), which is the gold standard for collecting incidence and survival data at the population level [46-51]. The absence of a PBCR means that cancer statistics are often derived from hospital-based registries, which only capture cases presenting to major referral centers and thus systematically underestimate the true burden, especially for rural populations and those with limited access to healthcare [48,50].

Even where hospital-based registries exist, their coverage is limited to a small fraction of the population, typically urban and peri-urban areas. Rural populations, internally displaced persons, and refugees are often excluded, leading to significant underestimation of cancer incidence and mortality [48-50]. The lack of unique patient identifiers and imprecise population denominators further complicate the calculation of accurate rates [48-50]. Additionally, the absence of legislation mandating cancer reporting and the lack of integration with national health information systems result in incomplete case ascertainment and poor follow-up for vital status [49,51].

Decades of conflict and political instability have severely undermined Afghanistan’s health infrastructure, impeding the establishment and maintenance of cancer registries. Armed conflict disrupts registry operations, leads to loss of trained personnel, and causes interruptions in data collection and reporting [47,49]. The mobility of refugees and internally displaced populations further complicates case tracking and follow-up [47,49].

Reliable cancer mortality estimates require robust vital registration systems capable of accurately recording causes of death. In Afghanistan, vital registration is incomplete, and death certification is often lacking or inaccurate. Many deaths occur outside the formal healthcare system and are not registered, while those that are registered may be miscoded or assigned “garbage codes” that obscure the true cause of death [50]. The lack of mortality data forces reliance on modeled estimates using regional averages or historical data from other countries, which introduces further uncertainty and potential bias [38,50].

Technical and human resource constraints further limit registry operations. Afghanistan faces a severe shortage of trained registry staff, limited financial resources, and inadequate information technology infrastructure. Most data collection remains paper-based, which is inefficient, prone to errors, and difficult to scale [51]. The lack of capacity for data analysis and dissemination further limits the utility of registry data for cancer control planning and policy development [38,51].

As of late 2025, there have been no documented new developments, initiatives, or pilot screening programs in cancer registry or surveillance systems in Afghanistan since 2023. The most recent regional review, which included direct contributions from Afghan experts and policymakers, confirms that Afghanistan’s cancer data are still derived almost exclusively from hospital-based case series in major urban centers and from extrapolations using regional averages or historical data from neighboring countries [1,52]. The absence of a PBCR means that Afghanistan cannot generate reliable, population-level incidence, survival, or mortality statistics for any cancer type [1].

Table 3 provides an overview of the current status of cancer registry and screening initiatives in Afghanistan, highlighting the urgent need for investment in registry and screening infrastructure.

**Table 3:** Current status of cancer registry and screening infrastructure in Afghanistan PBCR, population-based cancer registry

Initiative type	Status in Afghanistan (2023-2025)	Notes/details	References
PBCR	Not established	No PBCR; data modeled from regional averages; no new initiatives since 2023	[1-3]
Hospital-based/pathology registry	No new developments	Existing hospital-based data in Kabul; no expansion or upgrades since 2023	[1,3]
Digital registry integration	Not implemented	No integration with digital health systems; paper-based data collection	[1-3]

## Conclusions

Afghanistan faces a formidable cancer burden compounded by severe healthcare infrastructure deficits, resource constraints, and sociocultural barriers that profoundly limit the feasibility and effectiveness of cancer screening programs. This narrative review demonstrates that population-wide, high-technology screening programs are neither feasible nor cost-effective in Afghanistan’s current context. Instead, evidence-based, pragmatic strategies focused on opportunistic cervical cancer screening with VIA, early diagnosis initiatives for breast cancer, and integration with existing primary care and maternal health services offer the most realistic path forward. Establishing a comprehensive PBCR and strengthening vital registration systems are critical prerequisites for accurate burden estimation and evidence-based policy development. International support, sustainable funding, and political commitment are essential to translate these recommendations into meaningful improvements in cancer outcomes for the Afghan population.
